# The Eating and Cooking Healthy (TEACH) Kitchen: A Research Protocol

**DOI:** 10.21633/jgpha.6.2s20

**Published:** 2016

**Authors:** Sashia White, Roberto Alva-Ruiz, Lucia Chen, Jason Conger, Christopher Kuang, Cameron Murphy, Najeah Okashah, Eric Ollila, Selina A. Smith, Benjamin E. Ansa

**Affiliations:** 1 Medical College of Georgia, Augusta University, Augusta, GA; 2 Institute of Public & Preventive Health, Augusta University, Augusta, GA; 3 Department of Family Medicine, Medical College of Georgia, Augusta, GA

**Keywords:** nutrition, diet, cooking, chronic disease, diabetes mellitus, hypertension, hyperlipidemia

## Abstract

**Background:**

Diet-related chronic diseases, such as diabetes mellitus, hypertension, and hyperlipidemia have affected millions of individuals, resulting in disease-related complications and mortality. Strategies that may improve the outcome of chronic disease management include modification of lifestyle risk factors such as unhealthy diets. TEACH Kitchen is an experiential education program related to community nutrition, the goal of which is to teach patients management of chronic disease through dietary change.

**Methods:**

Adults (n=144) ≥18 years old and their children (n=144) 7–17 years old will complete four 2-hour sessions. Components of each session will include brief nutrition education (20 min), an interactive cooking session (1 hr), and after-dinner discussion (40 min). Pre- and post-session questionnaires will be administered to all participants for self-reported demographics, knowledge, attitude, and beliefs about healthy nutrition. Medical records will be used to collect information about adult participants’ demographics and clinical indicators (hemoglobin A1c, lipid profile, blood pressure, weight, height, and body mass index [BMI]). Descriptive analyses will be performed to determine socio-demographic characteristics using frequencies and proportions for all categorical data, and means for continuous variables. T-tests and multiple logistic regression analysis will be accomplished to compare the differences in means.

**Results:**

Differences in the pre- and post-session knowledge, attitude, and beliefs related to healthy eating will be evaluated for adults and children. The anticipated outcomes include enhanced education promoting healthy eating in the community, prevention of chronic disease complications related to poor diet, and prevention of obesity-related chronic diseases in children.

**Conclusions:**

Enhancement of chronic disease management among patients, and the prevention of obesity among children, can be accomplished through healthy cooking and diet.

## INTRODUCTION

According to the Centers for Disease Control & Prevention (CDC), six of the seven leading causes of death in the U.S. are chronic diseases. Almost half of all US adults (117 million people) have one or more chronic health condition, and one of four have two or more such conditions (Ward et al., 2014). Currently, 9.3% (29.1 million) of Americans and 9.9% (734,800) of Georgians are diagnosed with diabetes; 29% (70 million) of Americans and 35% (2.6 million) of Georgians have hypertension; and 4.2% of American adults and 4.1% of Georgians have coronary heart disease (Levi et al., 2015; Blackwell et al., 2014). More than 70 million U.S. adults have been diagnosed with hyperlipidemia, increasing their risk for cardiovascular disease and stroke (CDC, n.d.). In 2013, 38.1% of Georgia residents reported having high cholesterol (CDC, n.d.). Richmond County, Georgia, the site of the current proposed intervention, is ranked in the lowest 10% of Georgia’s 159 counties in terms of health indicators and surpasses the national benchmarks for obesity (CDC, n.d.).

Lifestyle behavior, such as unhealthy diet, is a risk factor for chronic diseases, including diabetes mellitus, hypertension, obesity, and hyperlipidemia. The high prevalence of such diet-related chronic diseases nationally and in Georgia highlights the need for nutrition-medical interventions for prevention and management of these conditions. Diets with excess sodium and cholesterol, along with inadequate amounts of fruits, vegetables, and whole grains, lead to poor health outcomes (Wartella et al., 2010). Counseling improves confidence in physician-directed changes in the patient’s nutrition regimen and diet-related chronic disease management (Franklin et al., 2013). Further, it is easier to change attitudes and knowledge regarding obesity and chronic disease prevention than to effect a change in physical attributes, such as body mass index (BMI), body fat, or waist circumference (Wood et al., 2008). Community cooking classes are a recently developed model for preventive medicine.

In conjunction with pharmacological therapy, management of chronic disease through modification of behavioral risk factors, such as diet, affords physicians and patients an opportunity to improve disease outcomes. The Eating and Cooking Healthy (TEACH) Kitchen, an experiential program for community nutrition education, aims to teach patients chronic disease management through diet, thus promoting healthy eating in the community. We hypothesize that providing nutrition education and cooking demonstrations will improve chronic disease management in the targeted population.

## METHODS

### Study design

TEACH Kitchen is a community intervention trial with a quasi-experimental (pre/post) design.

### Ethical consideration

The Institutional Review Board (IRB) at Augusta University approved the protocol for this study, and participants will be enrolled following consent.

### Study Population

For TEACH, two study populations are targeted. Recruitment goals include:

1. Adult males and females (n=144), ages 18 years and older diagnosed with diabetes, hyperlipidemia, obesity, or hypertension, and currently receiving care at Augusta University Healthcare (patients).
2. Children (n=144), ages 7–17 years, whose parents/legal guardians are participating in the current study (children). The children are patients with any of the listed chronic conditions.

Recruitment methods will be specific to the study population. Chronic disease patients will be referred by healthcare providers (e.g., Family Medicine, Internal Medicine, Cardiac Rehab) and student-run clinics (e.g., Clinica Latina, Equality Clinic, 8^th^ Street Clinic, Asian Clinic, FaithCare, Women’s Clinic). Flyers marketing the sessions will be placed in each clinic with contact information for a Lead Team member to allow patients to self-refer to TEACH. Children of patients will be recruited by word of mouth by participating patients. Participating adults will be encouraged to bring their children to the sessions. Assent will be obtained from the children who volunteer for the study.

### Procedures

With guidance from a chef and dietitian, TEACH investigators have developed a series of nutrition education seminars and cooking sessions focused on diabetes, hyperlipidemia, obesity, and hypertension (Table 1). For each chronic disease, a series of four 2-hour sessions will be presented. Each session includes: *Brief nutrition education* (20 minutes) related to the specific chronic disease (food and its nutritional content and foods to include, exclude, and limit) and reading nutrition labels. *Healthy cooking sessions* (1 hour) include food preparation techniques and healthy alternatives. *Guided after-dinner discussions* (40 minutes) include samples of prepared dishes, for which participants will complete a sensory evaluation of the appearance, taste, texture, aroma, and overall acceptability. They will also include focused discussions and review of nutrition education and the cooking sessions and implications for chronic disease management.

Methods for TEACH include educating chronic disease patients and their children. Each weekly session is 2 hours in length for 4 weeks. Participants are allowed to attend more than one 4-week block. Procedures for patients and their children are described below.

1. *Brief nutrition education* (20 minutes); specific disease (food and its nutritional content, foods to include, exclude and limit); reading nutrition labels – e.g., sessions - 1^st^ on disease, 2^nd^ on reading food labels
2. *Healthy cooking sessions* (60 minutes): food preparation techniques
3. *Guided after-dinner discussions* (40 minutes): Portion size, price, cost, healthy shopping techniques, meal planning in the context of cooking, making smart goals (developing core nutrition core competencies), and cooking techniques.
4. *Medical record extraction* (demographics include age, ethnicity, insurance status, gender, marital status, education; clinical indicators include hemoglobin A1c, lipid profile, blood pressure, weight, height, BMI; and other related health conditions) for adults.
5. *Health* c*ommunication* specific to adults and children.
6. *Surveys: B*aseline and post-intervention surveys, specific to adults and children. The questionnaires were developed by the TEACH facilitators and staff of the Institute of Public and Preventive Health at Augusta University, using modified versions of validated questionnaires available for use.
7. *Incentives:* A $25 gift card will be given to each adult at the completion of each 2-hour session. If a participant attends all 4 sessions, this would be a total of $100 in gift cards. Kohl’s Healthy Kids Kitchen incentives (a T-shirt, a chef’s hat, a spatula, and a measuring cup) will be given to each child at the completion of each 2-hour session.

### Data collection

Data will be collected by use of the following methods: self-administered questionnaire at baseline and post-session, sensory evaluation, and medical record extraction. Data will be collected and entered by the TEACH Kitchen team on tablets via the internet. Research Electronic Data Capture (REDCap) will be used to collect data and to generate an audit trail for tracking data manipulation and user activity. To ensure participants’ confidentiality, non-personal identifiers, assigned to each individual, will be used on all study documents.

Baseline and post-assessment surveys will be conducted for both patients and children. The baseline questionnaires will be administered to all participants, and the post-training questionnaires will be administered immediately following the fourth 2-hour session only to those participants who complete all four sessions. The survey provides self-reported characteristics for both patients and children, including demographics (i.e., ethnicity, age, education, marital status, religious preference, employment, income, and insurance status), as well as knowledge and attitudes towards nutrition and food servings. The children’s survey is modified to a ‘kid-friendly’ version. Participants will be considered to have completed the study after completion of 4 sessions. Participants who choose to withdraw from the study can do so without penalty and will be allowed to continue with the sessions.

### Statistical analyses

Descriptive analyses will be performed to determine the socio-demographic characteristics, using frequencies and proportions for all categorical data, and means for continuous variables. T-tests and multiple logistic regression analysis will be performed to compare the differences in means.

## RESULTS

The differences in participants’ pre- and post-session knowledge, attitude, and beliefs related to healthy eating will be assessed. The anticipated outcomes include enhanced education on promoting healthy eating in the community, prevention of chronic disease complications related to poor diet, and prevention of obesity-related chronic diseases in children.

## DISCUSSION

Lifestyle behaviors that include a healthful eating pattern are factors in reducing the disease burden associated with diet-related chronic disease (e.g., diabetes mellitus, hypertension, obesity, and hyperlipidemia). In response to a chronic disease diagnosis, patients often struggle to alter their lifestyle (Orzech, 2013). Changing cooking components, often part of nutritional interventions, are more effective than nutrition education (knowledge-, attitude-, and awareness-centered approaches) alone (Curtis et al., 2012).

Experiential cooking and nutrition education programs may be effective in improving nutrition in communities (Jarpe-Ratner et al., 2016). Dietary research tends to focus on selected outcomes, such as heart health, obesity, or diabetes, and these diets (e.g., for management of diabetes, hypertension, or hyperlipidemia) are not necessarily synonymous. Thus, nutrition education is generally specialized for individual populations, depending on their risk of certain diseases (Raber et al., 2016). TEACH Kitchen is an experiential community nutrition education program with a goal of teaching patients and their children chronic disease management through dietary change.

Unbalanced consumption of foods high in energy (sugar, starch, and/or fat) and low in essential nutrients contributes to energy excess, overweight, and obesity (World Health Organization, 2003). Among adults, consumption of fast food is associated with lower diet quality and obesity (Bowman et al., 2004). The amount of energy consumed in relation to the amount expended, and the quality of food are determinants of nutrition-related chronic disease. Food prepared at home provides fewer calories per eating occasion, and, on a per-calorie basis, provides less total and saturated fat, cholesterol, and sodium, and more fiber, calcium, and iron, compared to food prepared away from home (Guthrie et al., 2002). Among low-income women, increased frequency of consuming foods freshly prepared over a three-day period is associated with increased intakes of fruits and vegetables, protein, vitamin C, iron, zinc, and magnesium (Mclaughlin et al., 2003). To achieve best results in preventing and managing nutrition-related chronic diseases, strategies and policies should recognize the essential role of good nutrition. Cooking interventions that include hands-on food preparation show promise as a strategy for improving diet, positive food choices, and health outcomes as well as altering psychosocial factors, including food-related preferences and attitudes (Hersch et al., 2014; Reicks et al., 2014; Jarpe-Ratner et al., 2016). TEACH Kitchen is designed to promote healthy cooking and dietary choices among chronic disease patients and children.

## CONCLUSIONS

The prevalence of diet-related chronic diseases is high, and lifestyle behaviors, including healthy dietary intake, are factors in managing such conditions. TEACH Kitchen aims to promote healthy cooking and nutrition among patients and to reduce the obesity trend among children.

## Figures and Tables

**Table 1:** The TEACH Kitchen sessions based on specific chronic conditions

	#	Title	Content	Objectives
**Diabetes**	1 2 3 4	Managing your diabetes Living the lower carb life Weight-to-go What can I eat?	Aspects of diabetes management to help improve blood sugar levels Ways to promote weight loss and improve health while limiting carbohydrates Non-diet approach to changing eating habits Practical solutions to meal plan, reading labels, and reading labels	Learn practical ways to overcome challenges for living with diabetes Develop a lower carb meal plan, including dining out and weight management strategies Adopt a lifestyle approach to diabetes management Individualize meal planned based on food preferences
**Hypertension**	1 2 3 4	Managing your blood pressure Living the lower sodium life Weight-to-go What can I eat?	Aspects of hypertension management to help improve blood pressure levels Ways to promote weight loss and improve health while limiting salt Non-diet approach to changing eating habits Practical solutions to meal plan, reading labels, and reading labels	Learn practical ways to overcome challenges for living with high blood pressure Develop a lower sodium meal plan, including dining out and weight management strategies Adopt a lifestyle approach to diabetes management Individualize meal planned based on food preferences
**Obesity**	1 2 3 4	Managing your weight Lifestyle change Weight-to-go What can I eat?	Aspects of weight management Ways to promote weight loss through a healthy diet Non-diet approach to changing eating habits Practical solutions to meal plan, reading labels, and reading labels	Learn how being overweight affects overall health Develop a lower sodium meal plan, including dining out and weight management strategies Adopt a lifestyle approach to weight management Individualize meal planned based on food preferences
**Hyperlipidemia**	1 2 3 4	Managing your cholesterol Living the lower fat life Weight-to-go What can I eat?	Aspects of hyperlipidemia management to help improve blood lipid levels Ways to promote weight loss and improve health while limiting dietary fat Non-diet approach to changing eating habits Practical solutions to meal plan, reading labels, and reading labels	Learn differences between good and bad fats Develop a lower fat meal plan, including dining out and weight management strategies Adopt a lifestyle approach to hyperlipidemia management Individualize meal planned based on food preferences

## Statement of Student-Mentor Relationship

The lead author for this report, Sashia White, a medical student, participated in the Summer Public Health Scholars Program in the Institute of Public & Preventive Health at Augusta University. Dr. Benjamin E. Ansa, the senior author, served as her mentor.
